# Polyethylene glycol-conjugated HER2-targeted peptides as a nuclear imaging probe for HER2-overexpressed gastric cancer detection in vivo

**DOI:** 10.1186/s12967-018-1550-3

**Published:** 2018-06-19

**Authors:** Siao-Syun Guan, Cheng-Tien Wu, Chen-Yuan Chiu, Tsai-Yueh Luo, Jeng-Yih Wu, Tse-Zung Liao, Shing-Hwa Liu

**Affiliations:** 1 Institute of Nuclear Energy Research, Atomic Energy Council, Taoyuan, Taiwan; 2 Institute of Toxicology, College of Medicine, National Taiwan University, No. 1, Section 1, Jen-Ai Road, Taipei, 10051 Taiwan; 3 Institute of Food Safety and Health, College of Public Health, National Taiwan University, Taipei, Taiwan; 4 Department of Internal Medicine, Kaohsiung Municipal Hsiao-Kang Hospital, Kaohsiung Medical University Hospital, Kaohsiung Medical University, Kaohsiung, Taiwan; 5 Department of Medical Research, China Medical University Hospital, China Medical University, Taichung, Taiwan; 6 Department of Pediatrics, National Taiwan University Hospital, Taipei, Taiwan

**Keywords:** Gastric cancer, HER2 overexpression, HER2-targeted peptide, Nuclear medicine imaging

## Abstract

**Background:**

The human epidermal growth factor receptor 2 (HER2) involved proliferation, angiogenesis, and reduced apoptosis in gastric cancer (GC), which is a common target for tumor therapy. HER2 is usually overexpressed in more than 15% GC patients, developing a reliable diagnostic tool for tumor HER2 detection is important. In this study, we attend to use polyethylene glycol (PEG) linked anti-HER2/neu peptide (AHNP-PEG) as a nuclear imaging agent probe for HER2 detection in GC xenograft animal model.

**Methods:**

The HER2 expression of human sera and tissues were detected in GC patients and normal subjects. GC cell lines NCI-N87 (high HER2 levels) and MKN45 (low HER2 levels) were treated with AHNP-PEG to assess the cell viability and HER2 binding ability. The NCI-N87 was treated with AHNP-PEG to observe the level and phosphorylation of HER2. The MKN45 and NCI-N87-induced xenograft mice were intravenous injection with fluorescence labeled AHNP-PEG for detecting in vivo fluorescence imaging properties and biodistribution. The AHNP-PEG was conjugated with diethylenetriaminopentaacetic acid (DTPA) for indium-111 labeling (^111^In-DTPA-AHNP-PEG). The stability of was assessed in vitro. The imaging properties and biodistribution of ^111^In-DTPA-AHNP-PEG were observed in NCI-N87-induced xenograft mice.

**Results:**

The serum HER2 (sHER2) levels in GC patients were significantly higher than the normal subjects. The sHER2 levels were correlated with the tumor HER2 levels in different stages of GC patients. The AHNP-PEG inhibited the cell growth and down-regulated HER2 phosphorylation in HER2-overexpressed human GC cells (NCI-N87) via specific HER2 interaction of cell surface. In addition, the GC tumor tissues from HER2-postive xenograft mice presented higher HER2 fluorescence imaging as compared to HER2-negative group. The HER2 levels in the tumor tissues were also higher than other organs in NCI-N87-induced xenograft mice. Finally, we further observed that the ^111^In-DTPA-AHNP-PEG was significantly enhanced in tumor tissues of NCI-N87-induced xenograft mice compared to control.

**Conclusions:**

These findings suggest that the sHER2 measurement may be as a potential tool for detecting HER2 expressions in GC patients. The radioisotope-labeled AHNP-PEG may be useful to apply in GC patients for HER2 nuclear medicine imaging.

## Background

Gastric cancer (GC) is known as a highly lethal malignancy and a serious public health issue worldwide [1–3]. It is the fifth most frequent cancer and the third leading cause of cancer-related deaths of the world, with more than 980,000 new cases and 840,000 deaths occurring globally in 2013 [4]. Although the recent advances in diagnosis and therapeutic methods have been made, the advanced 5-year survival rates of GC patients are still less than 30% [5]. Moreover, it still remains difficult to cure as most GC cases are asymptomatic only until entering an advanced stage. Most of patients with adenocarcinoma of gastric cardia (AGC) present no effect on chemotherapy and chemo-resistance, which leads to poor survival and the limited therapeutic options [6]. Therefore, an early diagnosis intervention is important to provide early treatment for improving the prognosis of GC patients. Conventional white-light endoscopy (WLE) is a most commonly technique for many gastric diseases of the world in the last two decades [7]. However, to date, the application of WLE is limited to detect subtle mucosal alteration in flat lesions. There is also no standardized learning system, which could be commonly applied to endoscopic screening for early GC diagnosis [8]. Although histology of biopsy specimens is a gold standard method for diagnosing gastric lesions [9], it possesses a major disadvantage that premalignant lesions may be occurred by multiple occurrences, which may cause the missed diagnosis from random biopsy sampling in 20–30% of cancer cases [10, 11]. Therefore, development of noninvasive diagnostic methods such as nuclear molecular imaging with high specific biomarkers would be able to detect GC earlier and determine the therapeutic responsiveness in AGC patients.

Human epidermal growth factor receptor 2 (HER2) is a member of HER family of receptor tyrosine kinases [12, 13]. HER2 is a 185-kDa transmembrane protein, which comprises an extracellular ligand binding domain, a lipophilic transmembrane domain, an intracellular tyrosine kinase domain, and a carboxyl-terminal signaling domain [14, 15]. Unlike other HER family members, the high affinity ligand of HER2 has not been characterized [16]. It may be in an activated state constitutively or become active upon heterodimerization with other members of HER family such HER1 and HER3 [17, 18]. The homo- or hetero-dimerization of HER2 causes the autophosphorylation of the intracellular domain and triggers several downstream signaling cascades, including mitogen-activated protein kinases (MAPKs) pathway, the phosphatidylinositol-4,5-bisphosphate 3-kinase (PI3K)/AKT pathway for mediating cell proliferation, survival, differentiation, migration, and invasion, which are thought to be the signaling pathways responsible for the transforming potential of HER2-overexpressing cancers [19–24]. It has been recognized that overexpressed-HER2 leads to increased proliferation, angiogenesis, and reduced apoptosis occurring in various cancers: breast cancer [25–27], GC [28], ovarian cancer [29], salivary gland carcinoma [30], colon [31], bladder [32], lung [33], and esophagus [34]. Till now, only patients with breast cancer are routinely tested for the HER2 status. It is not yet the case for HER2 testing in GC patients. Hence, development of specific systemic HER2 targeting probe for detecting HER2 status is helpful for the optimal care of patients with advanced GC and the correct use of first-line drug therapy.

Trastuzumab, an anti-HER2 monoclonal antibody, has been employed as tumor-specific ligands for breast cancer and GC with HER2 overexpression in vitro and in vivo [35–37]. Trastuzumab has been approved by FDA for HER2-positive breast cancer therapy [37]. However, the intact antibodies as therapeutic or diagnostic molecules have limitations, including causing immune response, defects of resistance, low tumor penetration and high background noise [38, 39]. Compared to the shortcomings of macromolecules, utilization of tumor-targeting peptides as an efficient tumor imaging probe is a more valuable procedure [40–42]. The anti-HER2/neu peptide (AHNP, a 1.5 KDa cyclic peptide mimic of trastuzumab) possesses high HER2-specific affinity, which has been demonstrated to have similar activity to the intact antibody trastuzumab against tumor growth in vitro and in vivo [43–47]. Nevertheless, there is currently no effective AHNP-based nuclear medical agent for HER2-overexpressed gastric tumor imaging diagnosis in vivo. Herein, we conjugated diethylenetriaminopentaacetic acid (DTPA) and polyethylene glycol (PEG) with AHNP (DTPA-AHNP-PEG) for targeted imaging of HER2 positive GC in vivo. DTPA is a cyclic chelator, which can be radiolabeled with many radiometals such as ^111^In, ^64^Cu, and ^177^Lu [48]. PEG can increase the molecular mass, while reducing the rate of kidney excretion to prolong the blood circulating time of the drug [49]. Therefore, in this study, we aimed to develop a radiolabeled HER2 targeted agent for detecting HER2-overexpressed GC tumors in vivo.

## Methods

### Reagents

HS-PEG-NH_2_ (average molecular weight: 5 kDa), fluorescein isothiocyanate isomer I (FITC) and cyanine5.5 NHS ester (Cy5.5) were purchased from Sigma-Aldrich (St. Louis, MO, USA). Anti HER2/neu peptides, AHNP (sequence: FCDGFYACYMDV), AHNP-PEG, FITC-labeled AHNP-PEG (FITC-AHNP-PEG) were custom-made by AnaSpec (Fremont, CA, USA). The p-SCN-Bn-DTPA was obtained from Macrocyclics (Plano, TX, USA).

### Acquisition of the tissues and sera of GC patients

Clinical samples were acquired from Kaohsiung Medical University Chung-Ho Memorial Hospital (KMUH-IRB-980382), and written informed consent and approved by the Institutional Review Board from healthy subjects and patients with gastric cancer. The primary and contiguous normal tissues from GC patients were harvested after surgery. Histology and pathological grade and stage of tumors were followed the criteria from the 8th American Joint Commission on Cancer Staging Manual.

### Cell culture

MKN45, a human GC-derived cell line, was obtained from RIKEN BioResource center (Tsukuba, Ibaraki, JAPAN). NCI-N87, another human GC-derived cell line, was purchased from the Bioresource Collection and Research Center (Hsinchu, Taiwan). Cells were cultured in RPMI-1640 medium (Thermo Fisher Scientific, Waltham, MA, USA) added with 10% fetal bovine serum (FBS) (Thermo Fisher Scientific). Human primary stomach epithelium cells (HPSEC) was obtained from Cell Biologics (Chicago, IL, USA), which were maintained in Cell Biologics’ Cell Culture Medium supplemented with 10% FBS. All cells were under standard culture conditions (37 °C humid incubator with 5% CO_2_).

### Animals

The 8-week-old male BALB/c nude mice were purchased from BioLASCO (Taipei, Taiwan) and were housed under the controlled condition (a 12:12-h light: dark cycle at 22 ± 2 °C) and received rodent laboratory chow diet and drinking water ad libitum. All animal protocols were approved by the institutive ethical review committee and were in accordance with Taiwan regulations and National Institute of Health guidelines on the care and welfare of laboratory animals.

### Human serum HER2 assay

Human serum HER2 (sHER2) levels from 36 GC patients and 32 normal subjects were determined by a Human HER2 enzyme-linked immunosorbent assay (ELISA) kit (Thermo Fisher Scientific) according to the manufacturer’s instruction.

### Immunoblotting

Proteins of cells and tissues were extracted with the lysis buffer (the composition: 0.1% *sodium* dodecyl sulfate (SDS), 50 mM Tris–HCl (pH 8.0), 150 mM NaCl, 0.5% sodium deoxycholate, and 1% NP-40) and protein levels were determined by a Bradford protein Assay Reagent Kit (Bio-Rad, Hercules, CA, USA). The equal amounts of each protein samples were loaded in the 8% SDS polyacrylamide gel electrophoresis (SDS-PAGE). Immun-Blot^®^ polyvinylidene difluoride membranes (Bio-Rad) were used to transfer proteins from SDS-PAGE. After blocking with specific blocking buffer (Goal Bio, Taipei, Taiwan) for 2 min at room temperature, membranes were probed with primary HER2 antibody (1:2000) (Sigma-Aldrich) at 4 °C overnight. After washing membranes under standard washing procedure, membranes were probed with secondary antibody (dilution rate: 1:3000) (Sigma-Aldrich) at 4 °C for 1 h. The immunoreactive complexes were reacted with enhance chemiluminescence (Clarity™, Bio-Rad) and detected by using a LAS-4000 mini luminescent image analyzer (GE Healthcare; Uppsala, Sweden). Band densitometry was quantified by Multi Gauge v3.2 software (GE Healthcare).

### Histology and immunohistochemistry

Ten micrometer thick of GC tissues’ cryosections using a HM525 cryostat (Thermo Fisher Scientific) were mounted on gelatin-coated microscope slides and stained with hematoxylin and eosin for histological analysis. Cancerous lesions were performed by the methylene blue staining. The immunohistochemical analysis was performed on GC sections for HER2 and mki-67 staining with anti-human HER2 (1:200, Sigma-Aldrich) and anti-human mki-67 (1:200, Sigma-Aldrich) antibodies. The immunoperoxidase secondary detection system (Merck Millipore; Billerica, MA, USA) was applied to signal detection according to manufacturer’s protocols. Histology images were obtained with the Olympus DP70 microscope (Olympus, Tokyo, Japan) combined manufacturer’s digital imaging software (Olympus).

### Cell viability assay

Cell counting kit-8 (CCK-8, Sigma-Aldrich) was used to determine the cellular viability. Briefly, cells were cultured in 96-well plates at an optimized density under standard culture condition (37 °C, 5% CO_2_) for 16 h, and were then treated with AHNP-PEG and FITC-AHNP-PEG (0–100 μg/ml) for 24 and 48 h. Each well was added 10 μl of CCK-8 solution and incubated 1.5 h, and was measured the absorbance at 450 nm using a Bio-Rad microplate reader (Bio-Rad; Hercules, CA, USA).

### Flow cytometry analysis

MKN45 and NCI-N87 cells were cultured at an optimized density overnight, and then treated with 20 μg/ml FITC-AHNP-PEG for 2 h, while cells of competitive group were pre-treated with 20 μg/ml AHNP-PEG for 1 h. All cells were washed with PBS and collected for flow cytometric analysis using a BD Bioscience FACSCalibur Flow Cytometer (BD Bioscience, San Diego, CA, USA).

### Immunofluorescence staining

The AHNP-PEG binding assay of MKN45 and NCI-N87 was determined by immunofluorescence staining. Briefly, both cells were cultured on Merck Millipore Millicell EZ slide under standard cultured condition (37 °C, 5% CO_2_) overnight. After washing and fixing, fixed-cells were blocked with ThermoFisher Scientific SuperBlock Blocking Buffers for 30 min at room temperature and were then probed with FITC-AHNP-PEG (20 μg/ml) for 2 h at room temperature. The non-FITC AHNP-PEG (20 μg/ml) was as a competitor for competitive inhibition assay. The slides were counterstained with 0.2 μg/ml 4′,6-diamidino-2-phenylindole (Merck Millipore; Billerica, MA, USA) for 10 min at room temperature. The immunofluorescence-digital images were captured using a BX53 Olympus fluorescence microscope (Olympus) equipped with a charge-coupled device camera.

### AHNP-PEG and HER2 interaction assay

MKN45 and NCI-N87 cells (1 × 10^6^ cells) were treated with 20 μg/mL FITC-AHNP-PEG with or without 20 μg/ml non-FITC AHNP-PEG at 4 °C for 4 h, and then were lysed using an immunoblotting lysis buffer with protease inhibitors cocktail (Hycell International, Taipei, Taiwan). After centrifugation (14,000 rpm, 10 min, 4 °C), the supernatant was probed with or without 2 μg/ml biotinylated-HER2 antibody (Novus Biologicals, Littleton, CO, USA)-presented streptavidin agarose beads (Sigma-Aldrich) at 4 °C overnight. The fluorescence of immunoprecipitates was detected by a Wallac 1420 VICTOR2™ Fluorescent ELISA reader (Perkin Elmer, Waltham, MA, USA).

### The HER2 fluorescence imaging and distribution in GC xenograft mouse model

Tumor formation was established by subcutaneously inoculating MKN45 and NCI-N87 cells (2 × 10^6^) into the flank of nude mice (n = 3 per each group) for 2 weeks. For HER2 imaging assay in vivo, the AHNP-PEG was fluorescently labeled by Cy5.5 and intravenously injected into tail vein of MKN45 and NCI-N87-xenografted mice, respectively. After injection for 6 h, an in vivo fluorescent imaging system (PerkinElmer) was used to capture images of the whole animal body, and all xenograft mice were then sacrificed and harvested the following organs for distribution fluorescent imaging detection: tumor, lung, spleen, stomach, brain, heart, liver, kidney, colon, and muscle.

### DTPA labeled-AHNP-PEG synthesis and measurement

For preparation of DTPA conjugated-AHNP-PEG (DTPA-AHNP-PEG), AHNP-PEG and p-SCN-Bn-DTPA (w/w 1:50) was soaked in sodium carbonate buffer at 25 °C for 8 h. The conjugated-compounds were then purified by Amicon ultra centrifuge filters (3 kDa) (Merck Millipore). For molecular weight measurement, AHNP-PEG and DTPA-AHNP-PEG were detected with the matrix-assisted laser desorption/ionization time-of-flight mass spectrometry (MALDI-TOF MS; Bruker Daltonics, Billerica, MA, USA). The spectra were processed using FlexAnalysis™ 3.0 software (Bruker Daltonics). The DTPA-AHNP-PEG conjugation efficiency with indium-111 (^111^In-DTPA-AHNP-PEG) was evaluated by instant thin layer chromatography (ITLC) (AR-2000 radio-TLC Imaging Scanner; Bioscan, Washington, DC, USA).

### The stability assay of DTPA-AHNP-PEG and ^111^In-DTPA-AHNP-PEG in vitro

For DTPA-AHNP-PEG stability assay, the freeze-dried DTPA-AHNP-PEG was deliquesced in aqueous solvent under different conditions (pH: 6.0, 7.4, and 8.0; 37 °C), and the variance of DTPA-AHNP-PEG levels for up to 6 days was then evaluated. Samples were harvested from the stock solution and analyzed by using a high performance liquid chromatography. For ^111^In-DTPA-AHNP-PEG stability assay, the ^111^In-labeled compounds were incubated in human, fetal bovine, and mouse serum for 144 h. The radiolabeling yields of ^111^In-DTPA-AHNP-PEG were determined by ITLC every 24 h.

### In vivo HER2 nuclear imaging

After subcutaneouly inoculating NCI-N87 cells (2 × 10^6^ cells) into male BALB/c nude mice for 2 weeks, mice were received ^111^In-DTPA, ^111^In-DTPA-AHNP, ^111^In-DTPA-PEG, and ^111^In-DTPA-AHNP-PEG via intravenous injection with 1 mCi of indium-111. Nano Single-photon emission computed tomography/computed tomography (NanoSPECT/CT; Mediso Medical Imaging Systems; Budapest, Hungary) was applied to detecting HER2 distribution at 1, 4, 24, and 48 h.

### Ex vivo bio-distribution study

For tissue bio-distribution assay, NCI-N87 cells were injected into the right hind limb via the subcutaneous manner. Mice were intravenously received ^111^In-DTPA, ^111^In-DTPA-AHNP, ^111^In-DTPA-PEG, and ^111^In-DTPA-AHNP-PEG [0.55–0.67 MBq (15–18 μCi)] after inoculation for 2 weeks. Mice were then sacrificed and the dissected organs were weighted at 1, 4, 24, and 48 h post-injection. The radioactivity of samples was determined by 1470 WIZARD gamma counter (PerkinElmer). Data points were corrected for radioactive decay. The percentage of injected dose per gram of tissue (%ID/g) was calculated.

### Statistical analysis

Data are presented as mean ± standard deviation, and the significant differences between experimental groups (*p* value < 0.05) were considered by the following statistical analysis via GraphPad Prism V5.01 software (GraphPad Software, San Diego, CA, USA). The relationship between GC patients and normal subjects were evaluated by using the Chi squared test and Fisher’s exact test. Groups more than two were determined by one-way analysis of variance (ANOVA) followed by post hoc analysis with Bonferroni’s test.

## Results

### The overexpressed HER2 levels in serum and tumor tissues of GC patients

**Fig. 1:**
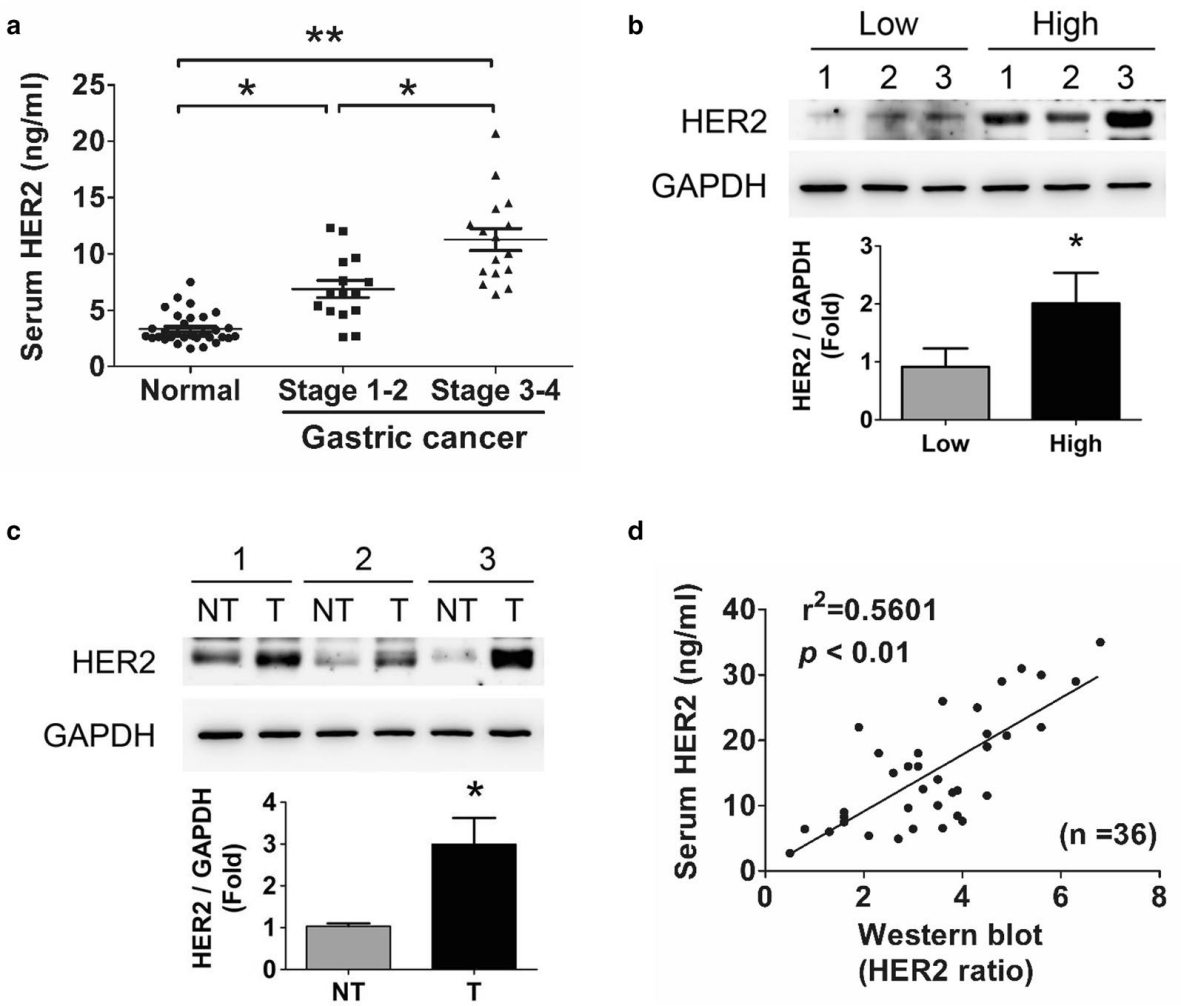
HER2 levels in sera and tumors of GC patients. **a** The changes of serum HER2 (sHER2) levels in healthy subject (n = 32) and GC patients with stage 1–2 (n = 15) and stage 3–4 (n = 21) detected using an ELISA assay. *P < 0.05, **P < 0.01. **b** The HER2 protein expression in gastric tumor tissues responding the sHER2 levels. The tissues were selected from three high- and three low-level sHER2 of GC patients. **c** The HER2 protein expression in gastric tumors (T) and non-tumor tissues (NT) from the individual GC patients with high sHER2 levels. In **b**, **c** the protein expression was determined by Western blotting and quantified by densitometry and normalized by GAPDH levels. Data are presented as mean ± SEM (n = 3). *P < 0.05, versus low sHER2 levels of GC patients (**b**); *P < 0.05, versus non-tumor (**c**). **d** The sHER2 levels were correlated with the HER2 protein expressions in tumor tissues of GC patients. *P* < 0.01, tumor HER2 versus sHER2

The sHER2 levels in patients with GC were significantly higher than the normal subjects. And the sHER2 levels at the stage 3–4 of GC patients were also significantly higher than that in patients with stage 1–2 of GC (Fig. 1a). The clinical characteristics of both GC patients and normal subjects were shown in Table 1. Moreover, the correlation between sHER2 and tumor HER2 in the individual GC patients was analyzed. The low-level sHER2 was defined as lower than the mean in the stage 1–2 of GC patients. On the contrary, the high-level sHER2 was defined as higher than the mean in the stage 3–4 of GC patients. The results of immunoblot analysis in tumors showed that the tumor HER2 protein expressions were higher in high-level sHER2 patients than in low-level sHER2 patients (Fig. 1b). In the individual patients with high-level sHER2, the higher HER2 protein expressions were observed in tumor tissues than in non-tumor tissues (Fig. 1c). As shown in Fig. 1d, the positively correlation between sHER2 levels and tumor HER2 levels (*P *< 0.05, r^2^ = 0.5601, Fig. 1d) was also observed. These results indicate that the sHER2 levels are capable of reflecting the tumor HER2 levels, which may be as a reference during cancer therapy.

**Table 1:** Clinical characteristics of normal volunteers and GC patients

Characteristics	GC patients (N = 36)		Normal subjects (N = 32)		*P* value
	N	%	N	%	
Age (years)					
≤ 50	14	38.89	18	26.25	0.224
> 50	22	61.11	14	43.75	
Gender					
Male	20	55.56	19	59.37	0.809
Female	16	44.44	13	10.63	
Differentiation					
1–2	19	52.78			
3–4	17	47.22			
TMN stage					
1–2	15	41.67			
3–4	21	58.33			
Tumor size (cm)					
≤ 5	17	47.22			
> 5	19	52.78			
Metastasis					
Yes	20	55.56			
No	16	44.44			

### Histological and immunohistochemical analysis of tumor and non-tumor tissues of GC patients

**Fig. 2:**
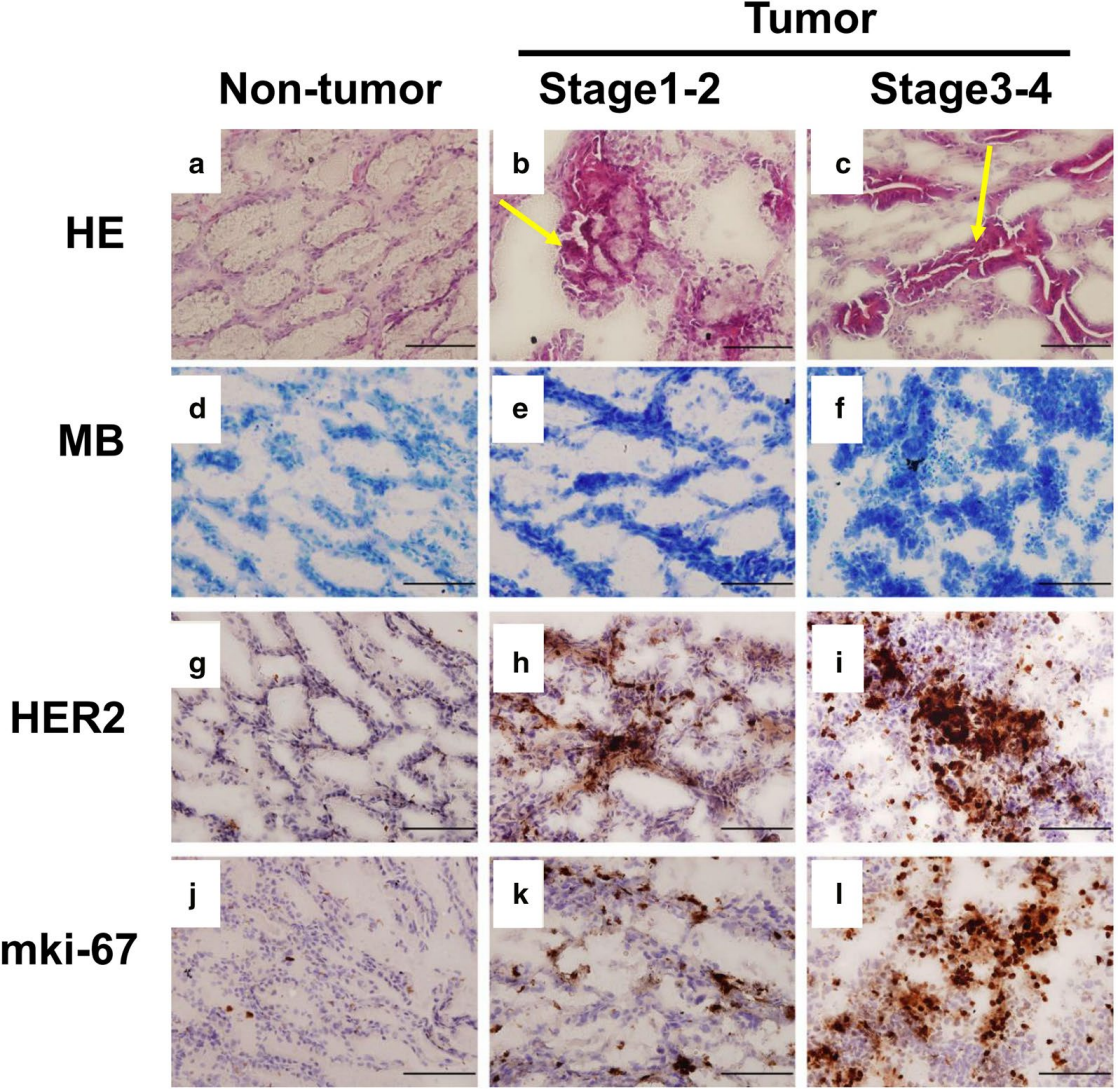
Histological and immunohistochemical analysis for HER2 expression in human GC. **a**–**c** Gastric tumor and non-tumor sections were stained with hematoxylin and eosin (HE). **d–f** Histopathological conformation of non-tumor and cancerous tumor tissues of gastric specimen were stained with methylene blue (MB). **g**–**l** Representative IHC staining images of GC sections for HER2 and mki-67 expressions. The nuclei of GC cells were clearly stained. HER2 (**g**–**i**) and mki-67 (**j–l**) were obviously expressed in stage 1–2 and stage 3–4 gastric tumor tissues, but weakly expressed in adjacent non-cancerous tissues. Magnification: ×400, scale bar: 50 μm

The morphological changes of tumor and non-tumor tissues of GC patients were observed by using hematoxylin and eosin stain. The strong staining of nuclei of epithelial cells was exhibited in the mucosa and stroma of GC tumor compared to non-tumor tissue (Fig. 2a–c). To confirm the cancerous tissue, the methylene blue staining was performed. As shown in Fig. 2d–f, the degree of deterioration in GC tumor at stage 3–4 was significantly stronger than in GC tumor at stage 1–2 and non-cancerous tissue. To investigate the distribution of HER2 in clinical specimens, the immunohistochemical assay was performed. As shown in Fig. 2g–i, HER2-positive staining was obviously presented in the cancerous gastric mucosa. No staining was observed for HER2 in human non-cancerous gastric mucosa. Moreover, the expression of mki-67, a proliferation marker, in GC tumor at stage 3–4 was higher than in GC tumor at stage 1–2 (Fig. 2j–l).

### Effects of AHNP-PEG on cell viability and HER2 binding in HER2-overexpressed GC cells

**Fig. 3:**
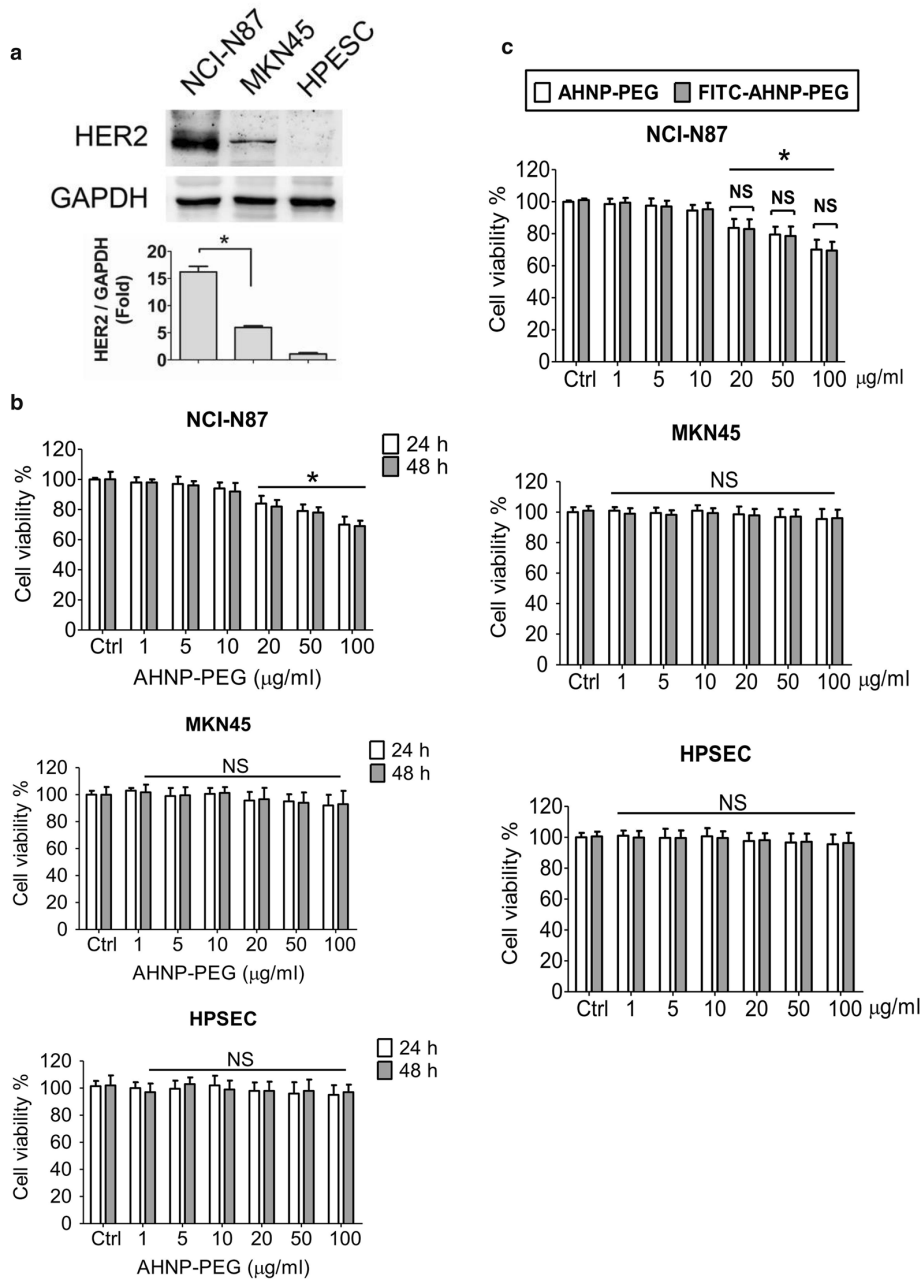
AHNP-PEG decreased cell viability in HER2-overexpressed GC cells. **a** The protein expressions of HER2 in GC cells (NCI-N87 and MKN45) and human primary stomach epithelium cells (HPSEC) were shown. Protein expressions were determined by Western blotting and quantified by densitometry and normalized by GAPDH levels. The data are presented as mean ± SEM (n ≥ 3). *P < 0.05, versus MKN45 cells. **b** The effect of AHNP-PEG on cell viability. The NCI-N87, MKN45 and HPSEC cells were treated with AHNP-PEG (0–100 μg/ml) for 24 and 48 h. Data are presented as mean ± SEM (*n *= 5). *P < 0.05, versus control; *NS* non-significant. **c** The effect on cell viability between AHNP-PEG and FITC-AHNP-PEG. The NCI-N87, MKN45, and HPSEC cells were treated with AHNP-PEG and FITC-AHNP-PEG (0–100 μg/ml) for 24 h. Data were presented as mean ± SEM (*n* = 5). *P < 0.05, versus control; *NS* non-significant

We next investigated whether PEG-conjugated AHNP (AHNP-PEG) has the therapeutic potential on GC cells with high-level HER2 expression (NCI-N87 cells) or low-level HER2 expression (MKN45 and HPSEC cells). The protein expressions of HER2 in GC cells were shown in Fig. 3a. Moreover, AHNP-PEG significantly decreased the cell viability in NCI-N87 cells, but little or no effects on cell viability of MKN45 and HSPEC cells (Fig. 3b). FITC-AHNP-PEG had similar effect with AHNP-PEG on cell viability (Fig. 3c). There was no significant difference between AHNP-PEG and FITC-AHNP-PEG groups.

**Fig. 4:**
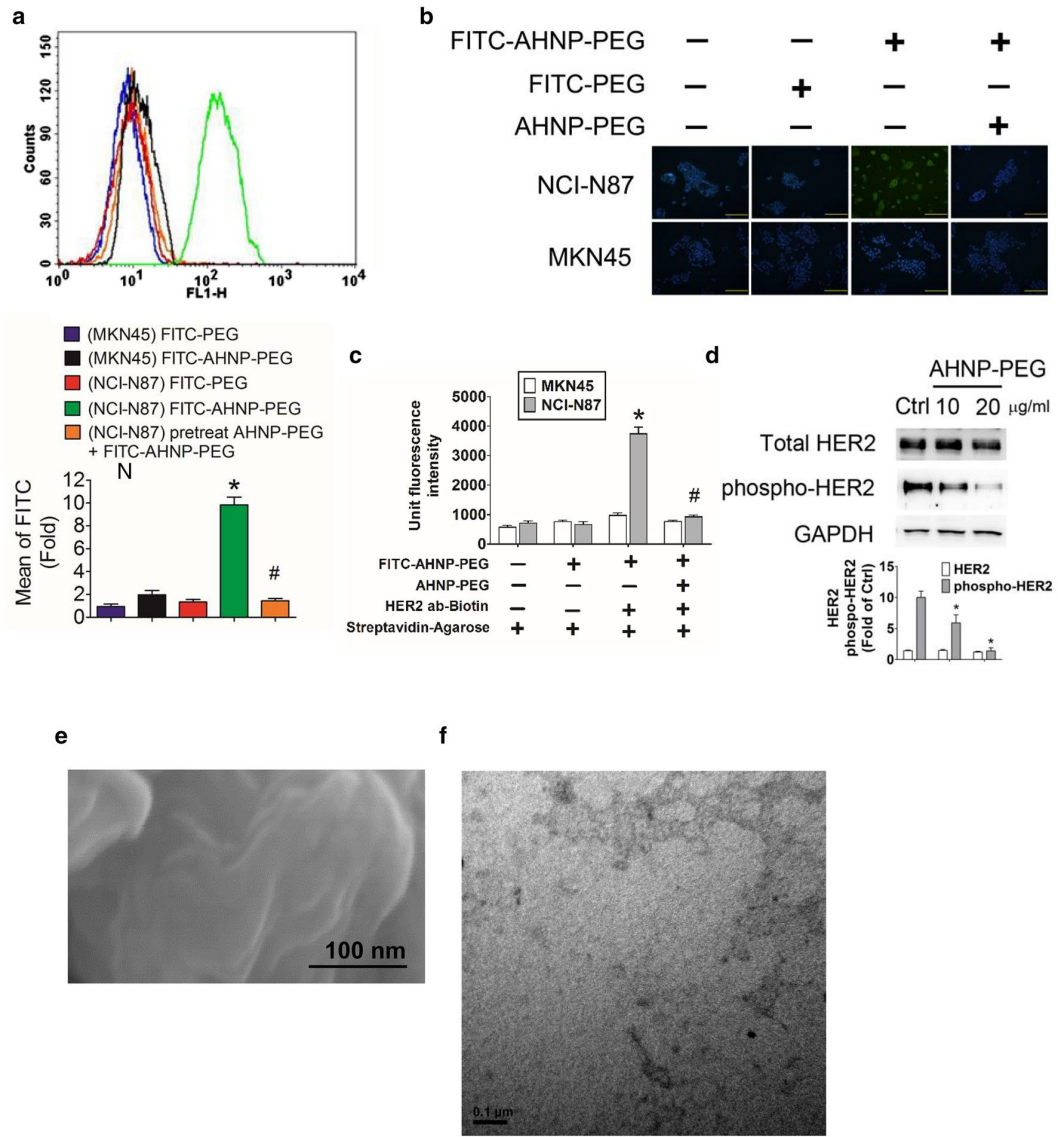
AHNP-PEG targeted to HER2 and reduced HER2 phosphorylation in GC cells. **a** AHNP-PEG binding assay was determined by flow cytometry in GC cells. MKN45 and NCI-N87 cells were treated with FITC-AHNP-PEG (20 μg/ml) for 2 h. In competitive group, NCI-N87 cells were pre-treated with AHNP-PEG (non-FITC; 20 μg/ml) for 1 h and then treated with 20 μg/ml FITC-AHNP-PEG for 2 h. Data are presented as mean ± SEM (n ≥ 3). *P < 0.05, versus FITC-PEG. ^#^*P* < 0.05, versus FITC-AHNP-PEG. **b** AHNP-PEG binding assay was observed by using fluorescent microscopy in GC cells. NCI-N87 and MKN45 cells were treated with FITC-AHNP-PEG (20 μg/ml) for 2 h. The nuclei were stained with 4′,6-diamino-2-phenylindole. Magnification: ×400, scale bar: 50 μm. **c** AHNP-PEG and HER2 interaction assay. NCI-N87 and MKN45 cells were treated with FITC-AHNP-PEG (20 μg/ml) for 4 h at 4 °C. The supernatant of lysates was incubated with or without biotinylated-HER2 polyclonal antibody (2 μg/ml) in the presence of streptavidin agarose beads at 4 °C overnight. The fluorescent signaling of immunoprecipitates was detected by using ELISA reader. The AHNP-PEG (non-FITC; 20 μg/ml) was pretreated for competitive inhibition assay. Data are presented as mean ± SEM (n ≥ 3). *P < 0.05, versus FITC-PEG. ^#^*P* < 0.05, versus FITC-AHNP-PEG. **d** The protein expressions of total and phosphorylated HER2 in AHNP-PEG-treated GC cells. NCI-N87 cells were treated with AHNP-PEG (10 and 20 μg/ml) for 24 h. Protein expressions were determined by Western blotting and quantified by densitometry and normalized by GAPDH levels. The data are presented as mean ± SEM (n ≥ 3). *P < 0.05, versus control group (Ctrl). **e**, **f** The structural and shape characteristics of the DTPA-AHNP-PEG by Scanning electron microscope (e; magnification: ×300 K, scale: 100 nm) and Transmission electron microscopy (f; magnification: ×100 K, scale: 100 nm)

In order to confirm the HER2 binding ability of AHNP-PEG, a flow cytometric analysis was performed in NCI-N87 and MKN45 cells. The fluorescence of FITC-AHNP-PEG was markedly and significantly presented in NCI-N87 cells, but not in MKN45 cells, compared to FITC-PEG treatment (Fig. 4a). Pretreatment with non-FITC AHNP-PEG significantly decreased the binding efficiency of FITC-AHNP-PEG in NCI-N87 cells (Fig. 4a). We further confirmed the binding ability of FITC-AHNP-PEG to HER2 in GC cells by using immunofluorescence. As shown in Fig. 4b, the fluorescence imaging was prominently increased in NCI-N87 cells, but not in MKN45 cells. The fluorescence imaging was significantly inhibited by pretreatment with non-FITC AHNP-PEG in NCI-N87 cells. To confirm that AHNP-PEG binds to the HER2 on the surface of GC cells, the immunoprecipitation assay was performed with HER2 antibody. As shown in Fig. 4c, FITC-AHNP-PEG could bind to HER2 in NCI-N87 cells, but not in MKN45 cells. The increased FITC-AHNP-PEG binding could be reversed by pretreatment with non-FITC AHNP-PEG. Moreover, we also observed that AHNP-PEG did not affect the total protein expression of HER2, but significantly decreased the phosphorylation of HER2 in NCI-N87 cells (Fig. 4d). Moreover, the structural and shape characteristics of the DTPA-AHNP-PEG were tried to observe by Scanning electron microscope (Fig. 4e) and Transmission electron microscope (Fig. 4f). Because of the small molecular size (~ 7 kDa), the substances produced image might not be obtained for structure and shape analysis.

### HER2 imaging in the tumors and organs of GC cells-induced xenografts in mice

**Fig. 5:**
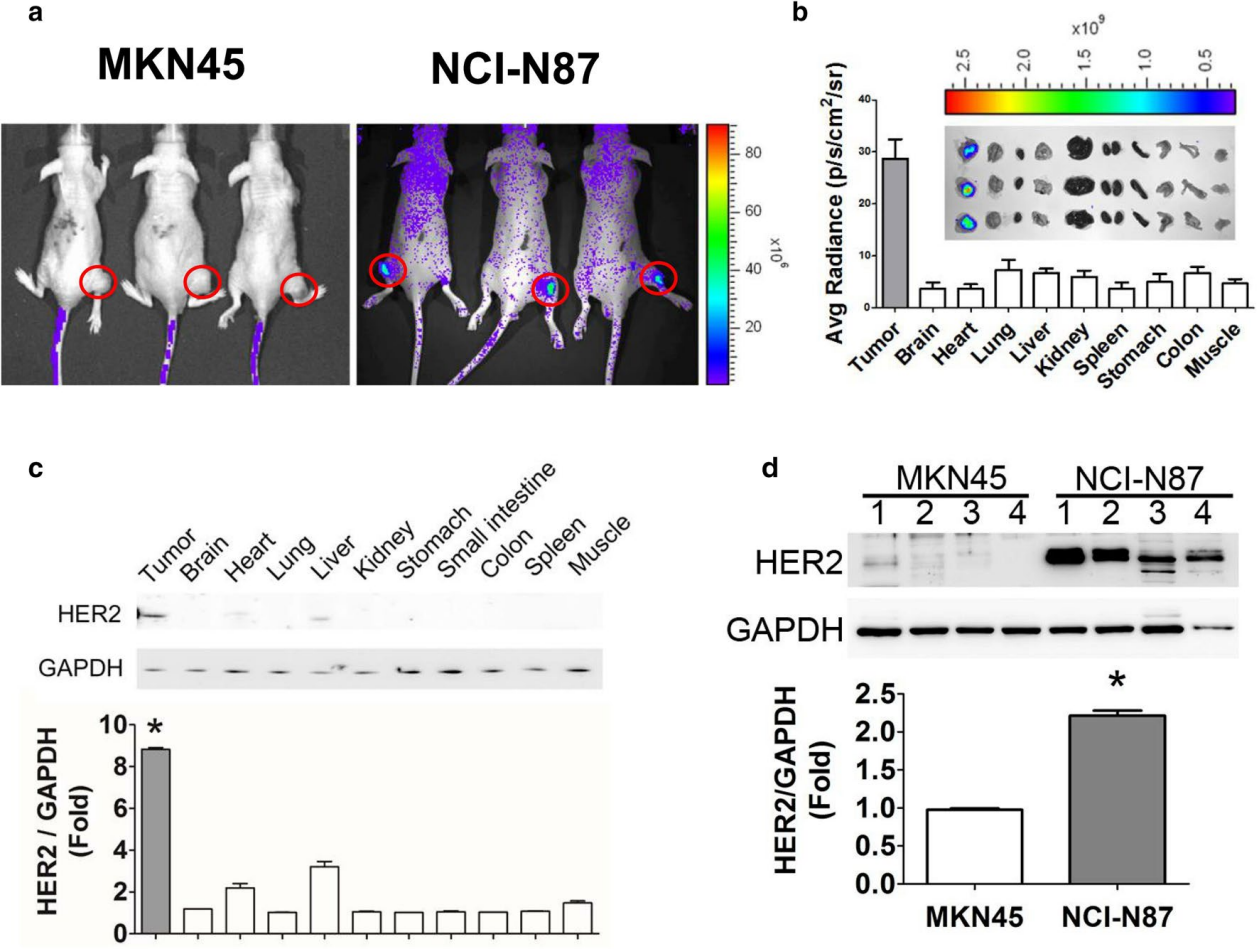
HER2 imaging in tumors and organs of NCI-N87- and MKN45-induced xenograft mice. **a** The HER2 imaging detection in NCI-N87 and MKN45 xenograft model. Both NCI-N87 and MKN45 cells (2 × 10^6^) were subcutaneously inoculated into the right flank of nude mice for establishing a HER2 high- and low-expressed xenograft model, respectively. The Cy5.5-conjugated AHNP-PEG was intravenous injected into mice for 6 h and detected by using IVIS. **b** The distribution of HER2 imaging in tumors and various organs (brain, heart, lung, liver, spleen, kidney, stomach, colon, and muscle) of NCI-N87 xenograft mice. The Top panel showed the HER2 imaging in various organs. Button panel showed the quantitation of fluorescent signals. **c** The protein expressions of HER2 in tumors and various organs of NCI-N87 xenograft mice. Protein expressions were determined by Western blotting. All data are presented as mean ± SEM (n = 3). **d** The HER2 expressions in tumors of NCI-N87- and MKN45-induced xenograft mice. Protein expressions were determined by Western blotting and quantified by densitometry and normalized by GAPDH levels. All data are presented as mean ± SEM (*n *= 3). *P < 0.05, versus MKN45-induced xenograft mice

To assess whether AHNP-PEG can be used as a diagnostic probe for GC detection, the GC xenograft models were established. The fluorescent signaling was significantly increased in the flank of NCI-N87 xenograft mice; but no fluorescent signaling was detected in MKN45 xenograft mice (Fig. 5a). We further observed the bio-distribution of HER2 in several organs in NCI-N87 and MKN45 xenograft mice. As shown in Fig. 5b, the fluorescent signaling for HER2 was significantly accumulated in tumor tissue compared to other organs in NCI-N87 xenograft mice. In addition, the HER2 proteins were overexpressed in tumor tissue compared to other organs in NCI-N87 xenograft mice (Fig. 5c), which was consistent with the results of HER2 bio-distribution imaging. The expression of HER2 in tumor tissue of NCI-N87 xenograft mice was higher than in tumor tissue of MKN45 xenograft mice (Fig. 5d).

### The ^111^In-DTPA-AHNP-PEG synthesis and stability assay

**Fig. 6:**
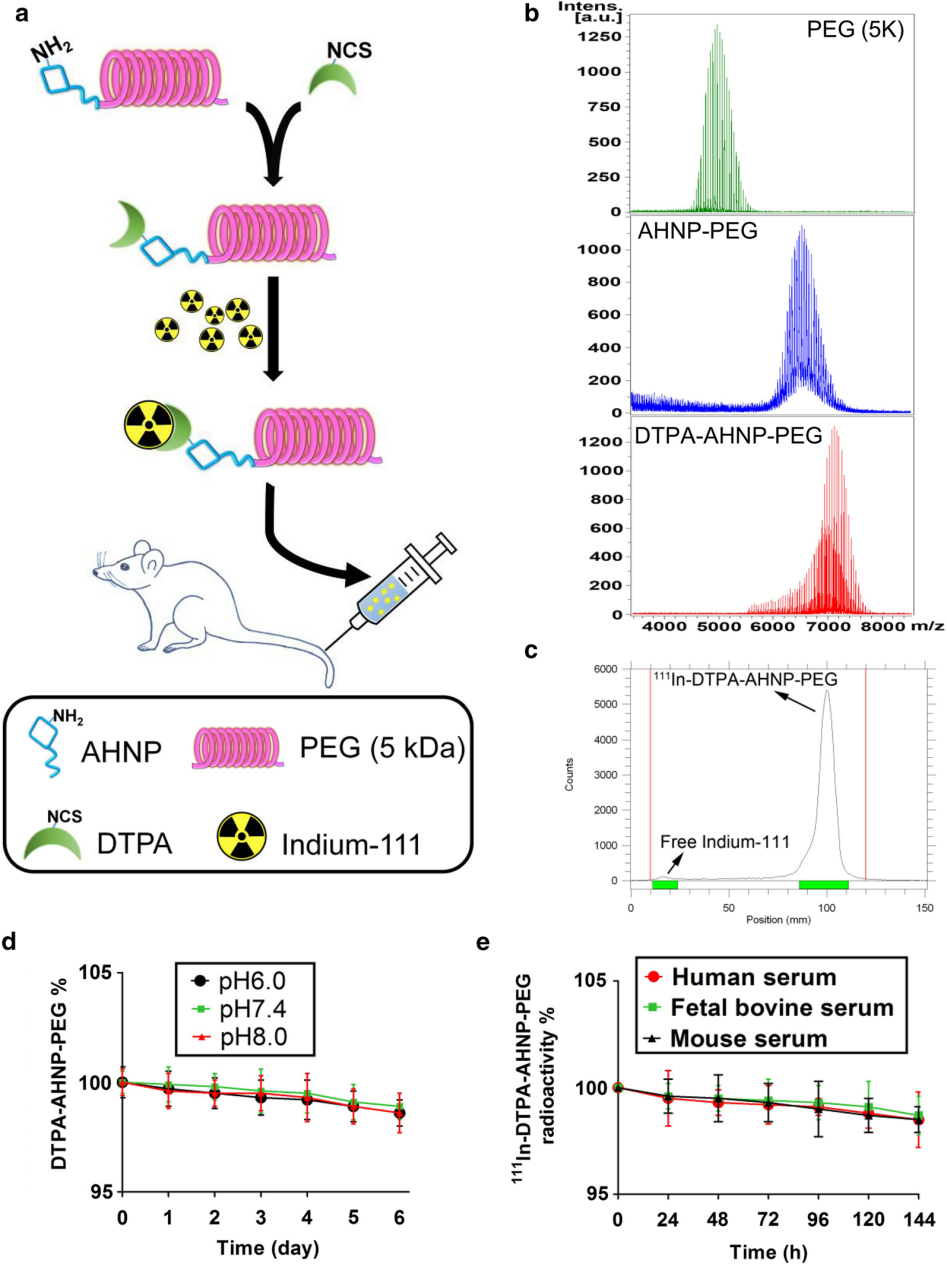
The ^111^In-DTPA-AHNP-PEG preparation and stability assay. **a** The flowchart of ^111^In-DTPA-AHNP-PEG synthesis was illustrated, where AHNP-PEG was conjugated with DTPA for indium-111 labeling. **b** The molecular weight assay for PEG, AHNP-PEG, and DTPA-AHNP-PEG. The molecular weights of PEG, AHNP-PEG and DTPA-AHNP-PEG were detected by using mass spectrometry. **c** The labeling efficiency of ^111^In-DTPA-AHNP-PEG. The DTPA-AHNP-PEG was labeled with indium-111 in PBS for 1 h. ITLC was performed to analyze the radio-labeling efficiency. **d** The stability of ^111^In-DTPA-AHNP-PEG in various pH buffers. The DTPA-AHNP-PEG was incubated at different buffer conditions (pH 6.0, 7.4, and 8.0) for 6 days at 37 °C and detected by HPLC. The results indicated that the levels of DTPA-AHNP-PEG was preserved more than 98% for 6 days. Data are presented as mean ± SEM (*n *= 5). *P < 0.05, versus day 0. **e** The stability of ^111^In-DTPA-AHNP-PEG in human, fetal bovine and mouse serum. The radioactivity of ^111^In-DTPA-AHNP-PEG was estimated in human, fetal bovine, and mouse serum for 144 h at 37 °C. The radioactivity was determined by ITLC. Data are presented as mean ± SEM (*n *= 5)

The above results suggest that AHNP-PEG may be used as a reliable probe for HER2 detection in HER2-overexpressed GC cells. DTPA has been extensively used as a metal chelator for isotope labeling in the development of radiopharmaceuticals. Therefore, the DTPA-conjugated AHNP-PEG (DTPA-AHNP-PEG) compound was synthesized for indium-111 labeling. The construct of the AHNP-based nuclear imaging agent is illustrated in Fig. 6a. During synthesis process, the DTPA-AHNP-PEG compound was analyzed using mass spectrom-etry to confirm the molecular weight of conjugation. As shown in Fig. 6b, DTPA were successfully conjugated with AHNP-PEG. The labeling efficacy of indium-111 onto the DTPA-AHNP-PEG was confirmed by instant thin layer chromatography and radioactivity detection, which indium-111 and indium-111-labeled DTPA-AHNP-PEG (^111^In-DTPA-AHNP-PEG) could be separated. As shown in Fig. 6c, the labeling efficacy of ^111^In-DTPA-AHNP-PEF was more than 99%. The levels of DTPA-AHNP-PEG were preserved more than 98% for 6 days in various pH conditions (pH 6.0, 7.4, and 8.0) (Fig. 6d). Moreover, the stability of ^111^In-DTPA-AHNP-PEG in serum of human, fetal bovine, and mouse was measured in a 144-h period. As shown in Fig. 6e, the radioactivity of ^111^In-DTPA-AHNP-PEG in these three species serum was preserved more than 97% for 6 days.

## ^111^In-DTPA-AHNP-PEG as a nuclear imaging agent for HER2-overexpressed GC tumor detection in vivo

**Fig. 7:**
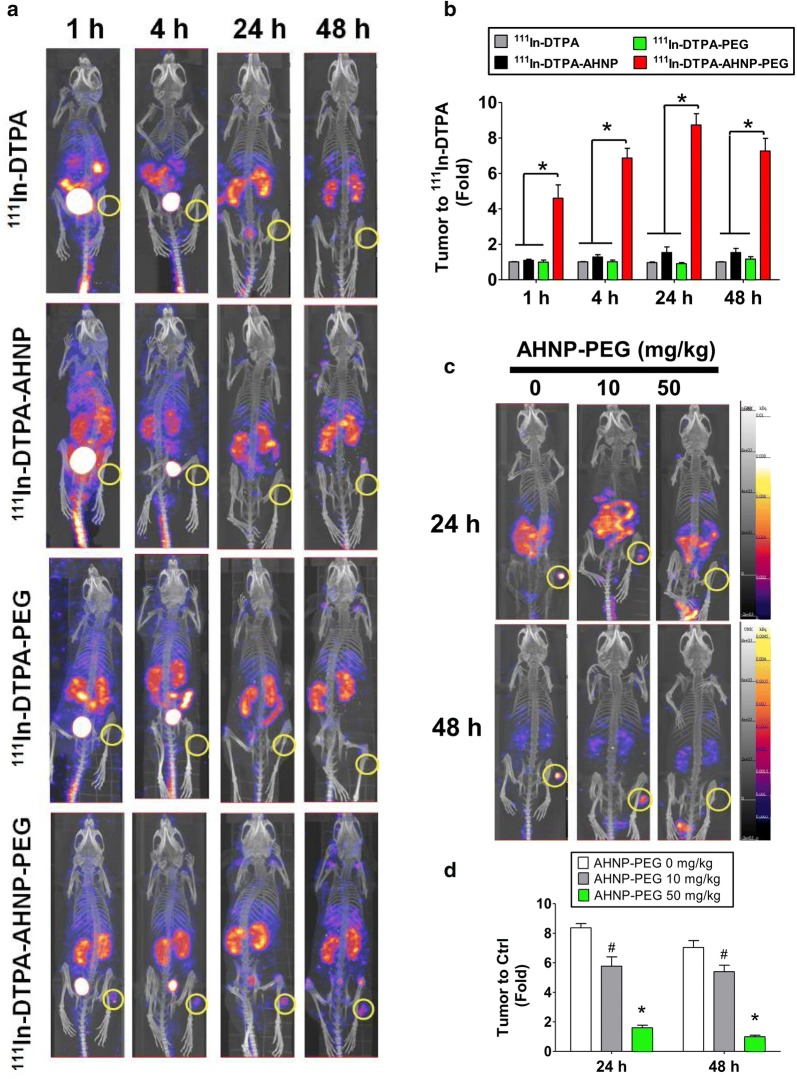
The nuclear imaging agent of ^111^In-DTPA-AHNP-PEG for gastric tumor detection in vivo. **a** The gastric tumor nuclear imaging analysis in NCI-N87-induced xenograft model. The ^111^In-DTPA, ^111^In-DTPA-AHNP, ^111^In-DTPA-PEG, and ^111^In-DTPA-AHNP-PEG were intravenous injected into mice for 1, 4, 24, 48 h and observed by using nanoSPECT/CT. **b** The oval-shaped labeling displayed the tumor site of gastric tumor xenograft mice for imaging quantification. Data are presented as mean ± SEM (n ≥ 3). *P < 0.05, versus ^111^In-DTPA, ^111^In-DTPA-AHNP, or ^111^In-DTPA-PEG group. **c** The blocking study of ^111^In-DTPA-AHNP-PEG in NIC-N87 tumor xenograft mice. After tumor xenograft animals were intravenous injected AHNP-PEG (0, 10, 50 mg/kg) for 4 h, ^111^In-DTPA-AHNP-PEG (1 mCi/per mouse) was intravenous injected into all mice for 24 and 48 h and observed by using nanoSPECT/CT. **d** The oval-shaped labeling displayed the tumor site of gastric tumor xenograft mice for imaging quantification. Data are presented as mean ± SEM (n ≥ 3). *P < 0.05, versus 10 mg/kg AHNP-PEG group, ^#^P < 0.05, versus 0 mg/kg AHNP-PEG group

**Fig. 8:**
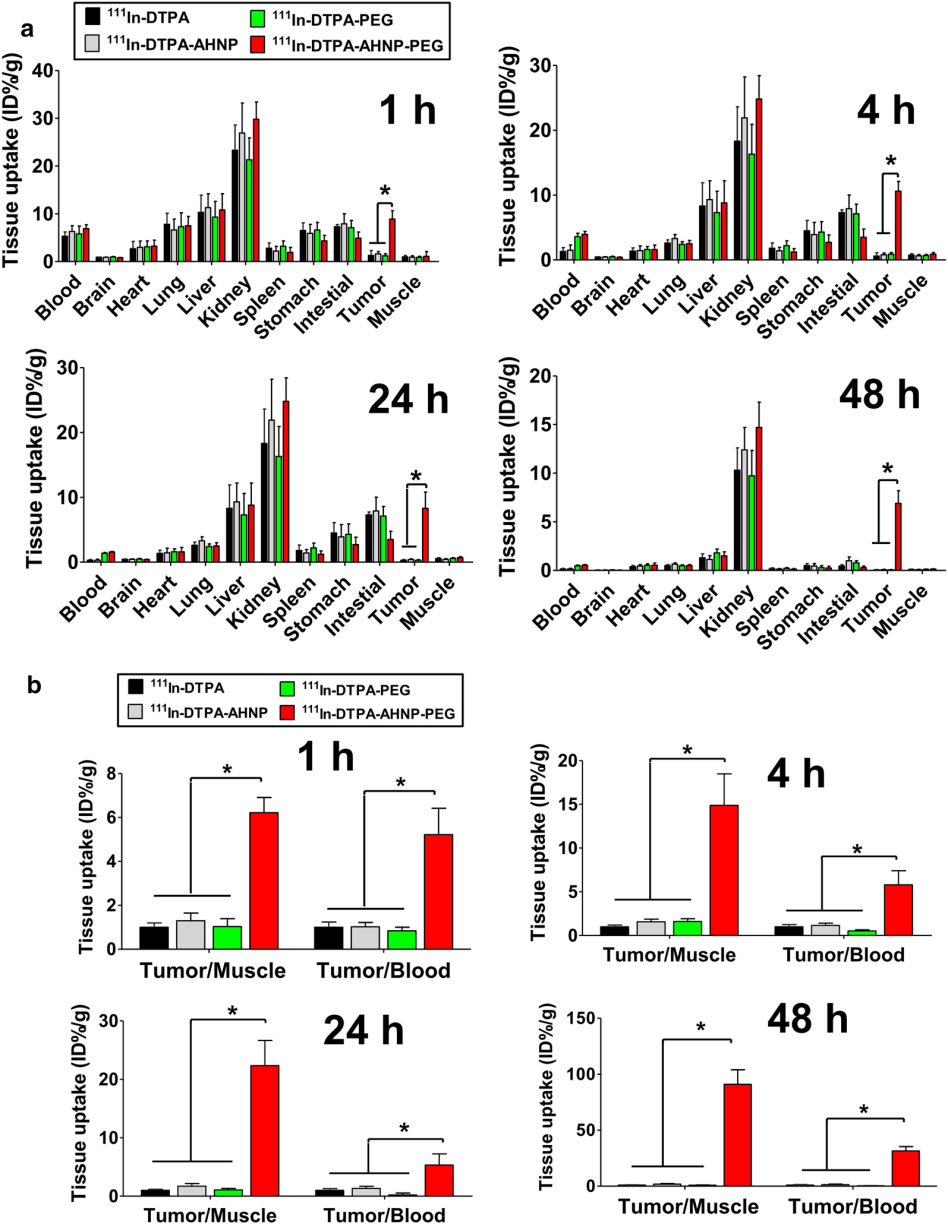
**a** Tissue biodistribution of ^111^In-DTPA-AHNP-PEG in gastric tumor xenograft mice. After ^111^In-DTPA, ^111^In-DTPA-AHNP, ^111^In-DTPA-PEG, and ^111^In-DTPA-AHNP-PEG intravenous injection for 1, 4, 24, and 48 h, NCI-N87-induced xenograft mice were sacrificed and acquired tumor, blood, brain, heart, lung, liver, kidney, spleen, muscle, stomach, and colon. The radioactivity was determined by a gamma counter. Values are expressed as the percentage of injected dose per gram organ (% ID/g). Data are presented as mean ± SEM (n ≥ 3). *P < 0.05, versus ^111^In-DTPA, ^111^In-DTPA-AHNP, or ^111^In-DTPA-PEG group. **b** Comparison of tumor-to-muscle (T/M) and tumor-to-blood (T/B) ratios among ^111^In-DTPA, ^111^In-DTPA-AHNP, ^111^In-DTPA-PEG, and ^111^In-DTPA-AHNP-PEG injected mice after 1, 4, 24, and 48 h injection. Data are expressed as mean ± SEM (n ≥ 3). *P < 0.05, versus ^111^In-DTPA, ^111^In-DTPA-AHNP, or ^111^In-DTPA-PEG group

In order to evaluate the efficiency of ^111^In-DTPA-AHNP-PEG in vivo, the HER2-expressed imaging in GC tumor was detected in NCI-N87-induced xenografts mice. After 1, 4, 24, and 48 h injection with ^111^In-DTPA-AHNP-PEG, the radionuclide signals in tumors were remarkable increased in treated group compared to control groups (^11I^n-DTPA, ^111^In-DTPA-AHNP, and ^111^In-DTPA-PEG) (Fig. 7a, b). The blocking study of ^111^In-DTPA-AHNP-PEG in NIC-N87 tumor xenograft mice was also tested and showed in Fig. 7c, d. We further investigated the bio-distributions of ^111^In-DTPA-AHNP-PEG in several organs of NCI-N87-induced xenografts. As shown in Fig. 8a, the tumor tissue presented higher radionuclide signal than other organs. We also evaluated the ratios of tumor-to-muscle (T/M) and tumor-to-blood (T/B) in ^111^In-DTPA, ^111^In-DTPA-AHNP, ^111^In-DTPA-PEG, and ^111^In-DTPA-AHNP-PEG-injected NCI-N87-induced xenografts. The ^111^In-DTPA-AHNP-PEG-treated group showed higher ratio in T/M and T/B compared to ^111^In-DTPA-treated group (Fig. 8b), indicating that ^111^In-DTPA-AHNP-PEG could target to HER2-expressed gastric tumor tissues and consequently improve the imaging efficacy.

## Discussion

In this study, we have found that the levels of sHER2 in patients with GC are significantly higher than the normal subjects and are correlated with tumor tissue HER2 levels. We also found that AHNP-PEG significantly inhibited cell growth in GC cells with high-expression HER2. The AHNP-PEG directly and specifically bound to HER2 on surface of HER2-overexpressed GC cells. In addition, we developed an agent as a nuclear imaging probe for HER2-overexpressed GC detection by using indium-111 labeled AHNP-PEG-conjugated DTPA. We found that ^111^In-DTPA-AHNP-PEG could successfully target to tumor site of HER2-overexpressed GC and captured tumor imaging in vivo.

Until now, chemotherapy is still the main treatment for GC patients with advanced stage [50, 51]. Many of patients are resistant to chemotherapy agents, including cisplatin [52, 53]. According to previous studies, HER2 has been found to induce tumor resistance to the current chemotherapy regimens in gastric and breast cancers [54, 55]. The overexpressed-HER2-activated downstream signals, such as the PI3K/AKT/epithelial-mesenchymal transition (EMT) pathway, has been suggested to be as the basis of HER2-mediated drug resistance [54–58]. Trastuzumab can target to HER2 for breast cancer therapy [59]. The patients with breast cancer received routine HER2 screening to decide whether carried out the process of trastuzumab treatment. For patients with GC, the frequency of HER2 overexpression was detected with a mean of 17.9% [60–62]. Therefore, the HER2 screening may need to be included in routine testing for GC to help the physicians to determine the best therapeutic strategy. The current standard HER2 screening method in clinical practice is assessed by immunohistochemistry (IHC) or fluorescent in situ hybridization (FISH) [63]. Although the characteristics of IHC are time-effective, low-cost and easy-to-perform for protein detection, it still has numerous limitations, including subjective interpretation of results, semi-quantitation, and variability dependent on fixation procedure, stain protocol, and antibody selection, which may result in low reproducibility and accuracy. Moreover, FISH is considered a gold standard method with highly sensitive and specific characteristics in detecting HER2 expression of tumor samples. However, it is not only expensive and time-consuming, but also needs a well-trained technologist. At present, most of the samples can be obtained through endoscopic gastric biopsy, but the unobvious cancerous tissue and low levels of HER2 expression in early stage of GC (stage 1–2) leads to a wrong diagnosis. In this study, we found that the sHER2 levels in GC patients was positively correlated with the tissue HER2 expression (r^2^ = 0.5601). Therefore, it may be used as a non-invasive blood screening method for a large-scale screening and reduce human error by the accurate analysis of instruments. In addition, we also developed a nuclear medicine molecular imaging agent for HER2 level detection. Therefore, both serum and nuclear medical image screening for HER2 may apply to detect the GC in early stage and monitor the progress of GC.

Nuclear medical imaging is a commonly used diagnostic tool in clinical. It has high sensitivity and can be immediately monitored for observing the changes at the lesion by physicians. It has been used for many disease diagnoses, including cardiovascular disease, bone, brain, and tumors. The usage of nuclear medical images is increasing yearly, which indicates the importance of this diagnostic tool and the potential for development. Nuclear medical imaging is characterized by the use of specific biomarkers as the subject at the lesion. The imaging reagent is designed as a target probe, which is conjugated with radioactive material for reaching the location of lesion via blood circulation. The radioactive material can be detected by special instrument for observing the location and distribution of lesions. The prostate-specific membrane antigen (PSMA) overexpression has been demonstrated to be a correlation between PSMA level and severity in prostate cancer [64, 65]. It has been further shown that the stage and grade of prostate cancer can be predicted by PSMA-based nuclear medical imaging [66]. In this study, we found that the expression of HER2 in GC was positively correlated with the tumor deterioration. The development of HER2 diagnostic agent ^111^In-DTPA-AHNP-PEG would be useful to obtain the information of distribution and levels of HER2 in tumors for determining the statue of GC. Both positron emission tomography (PET) and single-photon emission computed tomography (SPECT) are the main nuclear imaging systems in clinical practice. They are usually combined with computed tomography (CT) to acquire sequential images from both devices in the same session. Even though PET has highly resolution and sensitivity compared to SPECT, it still has numerous limitations, including required high-cost cyclotrons, high diagnostic cost, low market share, and limited half-life of radiopharmaceuticals. In recent years, the resolution of SPECT has gradually increased, which is getting closer to the PET. Near-infrared (NIR) fluorescence imaging system has been utilized in clinical practice for providing image-guided surgery, traumatic brain injury, and breast cancer detection [67–69], however, the limitations of tissue penetration and extra-cerebral contamination let it unable to visualize the deep tissue regions [68, 70]. In general, it seems difficult to replace nuclear medicine imaging for cancer patients at present.

The high specificity binding of molecules, such as antibodies, anti-peptides, and pharmacological inhibitors, could be a probe for targeting therapy and diagnosis. Trastuzumab is a specific HER2 antibody that is clinically used for HER2-overexpressing breast cancer therapy [71]. In addition, the high affinity HER2 binding antibody radio-labeled with zirconium-89 was also applied for diagnosing HER2-positive metastases in patients with HER2-negative primary breast cancer and advanced HER2-positive breast cancer [72, 73]. Even though antibody-based probe is the most frequently used for obtaining cancer imaging, the macro-molecule is no longer widely available for current in vivo imaging developments due to the size limitation and immunogenicity [74]. Although the modified antibody derivatives, such as mini-antibody, diabody, and scFv, improve the tumor tissue and blood vessel penetrability, the time to tumor localization, and the blood circulation clearance, but the protein engineering processes lead to the complicated manufacturing processes requirement [74]. In contrast, peptide agents are small molecules that have less immunogenicity, faster rates for tumor localization than antibodies, easy to synthesis, and contrast conjugation process. In this study, we have found that the AHNP peptides have the ability to bind to the target protein. AHNP is a small molecule of peptide-based imaging agent that could improve the physical property limitation of trastuzumab for clinical applications. In addition, the PEG-conjugated AHNP peptide solved the shortcomings of rapid excretion of imaging agent in vivo and enhanced the accumulation in tumor.

Nanoparticles are known to accumulate in the tumor site through enhanced permeability and retention effect. PEG-covered liposomal doxorubicin has been shown to increase tumor toxic effects on HER2 over-expression breast cancer in vitro and in vivo [75]. In addition to increasing the accumulation of tumor sites, the nanoscale drug carrier also accumulated in liver, spleen, and heart that may result in side effects. The quite large volume of liposome easily leads to phagocytosis by macrophage and accumulation in the reticuloendothelial system. Moreover, in order to increase the specificity of tumor binding for tumor therapy and diagnosis, the nanoparticles-conjugated tumor specific target probes have been considered. AHNP-conjugated iron oxide nanoparticles significantly increased the accumulation in tumor area after intravenous injection for 48 h in breast cancer xenograft mouse model [76]; however, the in vivo fluorescent imaging analysis has indicated that drugs still accumulate in other organs for up to 96 h. Yang et al. recently developed a dual-targeting hybrid nanoparticles system for GC detection [77]. The nanoparticles was made of biodegradable polymer and coated with a lipoid shell prepared by conjugating the AHNP peptides and *n*-hexadecylamine to the carboxyl groups of hyaluronic acid. In the study of Yang et al. the bio-distribution assay showed that the nanoparticles significantly accumulated not only in tumor area but in other organs, such as liver, lung, spleen, and heart. The long-term blood circulated nanoparticles may result in side effects during tumor therapy. In the present study, we found that the PEG-conjugated AHNP significantly accumulated in tumor site, but reduced the accumulation in other organs.

The previous studies reported that HER2 triggered the activation of downstream molecular pathway and caused cancer cell proliferation and invasion in HER2-positive cancers [78, 79]. Faltus et al. observed that the proliferation of HER2-overexpressed breast cancer cells could be inhibited by using siRNA to downregulate HER2 gene expression [80]. It has been found that the application of antibody-engineered multifunctional nanoparticles downregulates the protein expression of HRE2 on the cell surface of breast cancer cells [81]. Li et al. recently indicated that the biparatopic HER2-targeting antibody could significantly promote HER2 receptor clustering, internalization, and lysosomal degradation [82]. In addition to antibodies, AHNP has also been found to induce rapid HER2 receptor internalization and reduce HER2 expression in HER2-transfected mouse fibroblasts cells (NIH-HER2^+^) [83]. They showed that the dimeric peptide ligand AHNP_bivalent_ significantly induced about 18% reduction in surface HER2 density and enhanced cytotoxicity in NIH-HER2^+^ cells. However, the monomeric peptide ligand AHNP_monovalent_ did not cause HER2 internalization. It has been considered that the helical segments structure and receptor dimers stabilization are two possible mechanisms for peptide-induced receptor internalization. Interestingly, the current study observed that the HER2 receptor expression did not change, but phosphorylation of HER2 was significantly decreased after 24 h ANHP-PEG treatment in GC cells. Therefore, our results suggest that the AHNP-HER2 complex may decrease phosphorylation and interrupt the HER2 downstream signals to inhibit cell viability in GC cells. The AHNP-PEG might be designed as a theranostic agent for tumor diagnosis and therapy in the future.

## Conclusion

In this study, we demonstrated that the sHER2 levels are positively correlated with tissue HER2 expression in GC patients, suggesting that sHER2 may be a useful tool for detecting tissue HER2 expressions. AHNP-PEG specifically bound to HER2-overexpressed GC cells and inhibited cell proliferation in vitro. We successfully synthesized DTPA-AHNP-PEG with high stability and specific HRE2 targeting in vivo. It can be radiolabeled with In-111 for measuring and analyzing HER2 imaging by using nanoSPECT/CT in GC xenograft mice with HER2 overexpression. Therefore, ^111^In-DTPA-AHNP-PEG may be a potential nuclear imaging agent for diagnosis of HER2-overexpressed GC. The HER2 bound AHNP-based nuclear imaging may provide to monitor patients with a history of GC after surgery or drug treatment.

## Abbreviations

AGC: adenocarcinoma of gastric cardia
AHNP: anti-HER2/neu peptide
CCT-8: cell counting kit-8
CT: computed tomography
Cy5.5: cyanine5.5 NHS ester
DTPA: diethylenetriaminopentaacetic acid
ELISA: enzyme-linked immunosorbent assay
GC: gastric cancer
HER2: human epidermal growth factor receptor 2
HPLC: high performance liquid chromatography
HPSEC: human primary stomach epithelium cells
IHC: immunohistochemistry
IVIS: in vivo imaging system
ITLC: instant thin layer chromatography
PEG: polyethylene glycol
PET: positron emission tomography
PI3 K: phosphatidylinositol-4,5-bisphosphate 3-kinase
PBS: phosphate buffered saline
PSMA: prostate-specific membrane antigen
SDS: sodium dodecyl sulfate
sHER2: serum HER2
SPECT: single-photon emission computed tomography
TCMN: trastuzumab-conjugated multifunctional nanoparticles
T/B: tumor-to-blood
T/M: tumor-to-muscle
WLE: white-light endoscopy
